# Standard versus restricted fluid administration in preterm infants undergoing pharmacological treatment for haemodynamically significant patent ductus arteriosus

**DOI:** 10.1038/s41390-025-04497-w

**Published:** 2025-10-27

**Authors:** Angela Paladini, Vito D’Andrea, Anthea Bottoni, Giorgia Prontera, Velia Purcaro, Giulia Vanina Cantone, Giovanni Vento

**Affiliations:** Dipartimento Universitario Scienze della Vita e Sanità Pubblica, UOC di Neonatologia, Fondazione Policlinico Universitario Agostino Gemelli IRCCS—Università Cattolica del Sacro Cuore, Rome, Italy

## Abstract

**Background:**

Fluid management in patent ductus arteriosus (PDA) treatment is debated, with fluid restriction traditionally used to promote closure, though its impact on outcomes is uncertain.

**Methods:**

This retro-prospective cohort single-center study compared two cohorts of preterm neonates receiving pharmacological treatment for hemodynamically significant PDA (hsPDA). The 2021–2023 cohort followed an updated, higher fluid intake protocol based on neonatal weight and day of life (80–100 ml/kg/day for extreme preterm <1000 g on day 1), while the 2018–2020 cohort adhered to a more restrictive approach based on gestational age and day of life (70 ml/kg/day for extreme preterm ≤26 weeks on day 1) and fluid restriction from 5 to 20% during pharmacological treatment of hsPDA. Outcomes included PDA closure rates, growth parameters, and neonatal complications.

**Results:**

hsPDA closure rates and major complications (intraventricular hemorrhage, necrotizing enterocolitis, or chronic lung disease) were similar between groups. However, fluid-restricted neonates required more central catheter days, longer times to full enteral feeding, and had poorer growth at discharge (lower weight z-scores).

**Conclusions:**

Fluid restriction during pharmacological PDA treatment may adversely affect growth and clinical care without improving closure rates. A balanced, individualized fluid management strategy could enhance neonatal outcomes.

**Impact:**

- The article suggests that fluid restriction does not improve the response to pharmacological treatment for PDA, but could negatively influence growth of preterm infants before discharge, which might have long term repercussions.
- It highlights there is no benefit, but potential harm, of fluid restriction to promote PDA in this vulnerable population.
- The findings could guide changes in clinical practice, improving PDA management and potentially reducing complications for preterm infants who are highly sensitive to fluid imbalances.
- The study advocates for further clinical trials to explore the optimal hydration strategies in managing PDA, paving the way for future research that can refine and standardize treatment protocols.

## Introduction and background

The ductus arteriosus (DA) plays a critical role during fetal development. Under normal conditions, it closes spontaneously within the first few days after birth. However, especially in premature infants, this closure may fail, a condition that might potentially lead to volume overload of the left ventricle and systemic hypoperfusion. Failure of ductal closure occurs in up to 33% of very low birth weight (VLBW) neonates and up to 65% of extremely low birth weight (ELBW) neonates. The continued presence of a patent ductus arteriosus (PDA) can elevate the likelihood of developing intraventricular hemorrhage (IVH), necrotizing enterocolitis (NEC), and chronic lung disease.^1,2^ Despite the high incidence of this condition, there is still no universal consensus regarding the management and treatment of PDA.^3^

Optimal management for an hemodynamically significant PDA (hsPDA) is controversial. Pharmacological treatment for hsPDA has been proven to be effective in its closure, however, closing a PDA has not been definitively shown to improve overall outcomes in neonates. There is a large variability between centers in management and treatment of an hsPDA. The absence of clear benefits associated with PDA treatment has recently led to the adoption of a more conservative management strategy.^4,5^

Fluid management and its relation with PDA is still an open issue. High fluid administration in the first days of life, above 170 m/kg/dayon day 3 of life, has been related to an increased risk of PDA among infants p1250 g, as some studies recommended fluid restriction as a prophylactic tool in PDA but also a thrapeutic tool when a hemodynamically significant PDA is diagnosed, with the rationale of decreasing transductal shunt volume thereby facilitating closure and reducing pulmonary blood flow and hyperperfusion symptomatology.^6,7^ Despite evidence supporting fluid restriction in neonates with an already hsPDA remaining outdated, inconclusive, with no significant reduction in pulmonary overflow and concerns about the potential deterioration of regional blood flow, fluid restriction is used frequently.^8^

To date, questions regarding whether fluid restriction should be applied following the diagnosis of PDA or only in cases of hemodynamic significance, remain unanswered.^9^

The aim of this study was to investigate the efficacy of fluid restriction, in the medium- and long-term, when applied during the medical closure of PDA.

## Methods

This is a retro-prospective cohort single-center study, conducted in the third-level neonatal intensive care unit (NICU) of Fondazione Policlinico Universitario A. Gemelli IRCCS, Rome—Università Cattolica del Sacro Cuore.

The study analyzed two groups of preterm neonates undergoing pharmacological closure treatment for hsPDA: a prospective group (PG) studied during the years 2021–2023 characterized by no fluid restriction during treatment, and a second historical group (HG), born between 2018–2020 characterized by fluid restriction during treatment.

At the Fondazione Policlinico Universitario A. Gemelli NICU, from 2021, the internal protocol for fluid management was revised, and higher fluid intakes were implemented from birth, in line with the most recent ESPGHAN recommendations.^10^

The new recommended fluid intake (ml/kg/day) during the first days of life in neonates differs based on neonatal weight and day of life (Supplementary Table S1) :

(1) Preterm neonates >1500 g (day 1: 60–80, day 2: 80–100, day 3: 100–120, day 4: 120–140, day 5 and onwards: 140–160); (2) Preterm neonates 1000–1500 g (day 1: 70–90, day 2: 100–110, day 3: 110–130, day 4: 130–150, day 5 and onwards: 160–180); (3) Preterm neonates <1000 g (day 1: 80–100, day 2: 100–120, day 3: 120–140, day 4: 140–160, day 5 and onwards: 160–180).

In the same year, a standardized protocol for enteral nutrition was introduced, recommending the initiation of minimal enteral feeding (MEF) within the first 3–48 h of life with maternal milk or donor human milk for all VLBW neonates.

Before 2021, the internal protocol for fluid management (ml/kg/day) followed a more restrictive approach based on gestational age (Supplementary Table S2):

1. Gestational age >26 weeks (day 1: 60, day 2: 70, day 3: 80, day 4: 100, day 5: 120; day 6: 140, day 7 and onwards: 150);
2. Gestational age ≤26 weeks (day 1: 70, day 2: 80, day 3: 90, day 4: 110, day 5: 130; day 6: 140, day 7 and onwards: 150).

As well as fluid intake, the management of enteral feeding in preterm infants with hsPDA remains controversial, as robust evidence is lacking and randomized trials are limited. Given this uncertainty, our unit has adopted a cautious strategy, often maintaining trophic feeding during treatment.

The primary outcome was to evaluate the efficacy of fluid restriction in the response to pharmacological treatment of hsPDA by comparing the two groups.

The secondary outcomes were: anthropometric z-scores (weight, length, and head circumference) at birth and discharge; bronchopulmonary Dysplasia (BPD) at 36 weeks’ postmenstrual age; death at 36 weeks’ postmenstrual age or before discharge; severe IVH (grade 3 or 4 based on the Papile criteria);^11^ occurrence of air leaks, including pneumothorax (PTX); need for and duration of invasive respiratory support; duration of non-invasive respiratory support; duration of oxygen therapy; incidence of pulmonary hemorrhage, periventricular leukomalacia (PVL),^12^ retinopathy of prematurity (ROP) grade 3 or higher,^13^ NEC grade 2 or higher,^14^ sepsis (defined as a positive blood culture or suggestive clinical and laboratory findings leading to treatment with antibiotics for at least 7 days, despite the absence of a positive blood culture); days to achieve full enteral feeding; days of central line use; total in-hospital stay.

**Fig. 1:**
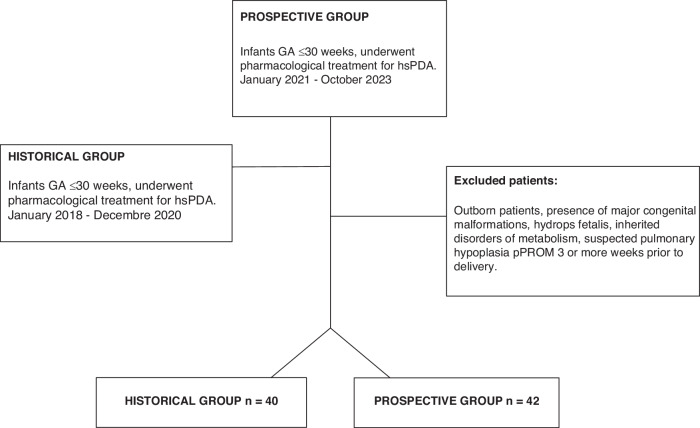
Flow diagram.

We enrolled all patients admitted to the NICU, with a gestational age ≤30 weeks, born between January 1, 2018, and October 31, 2023, who underwent pharmacological treatment for hsPDA and received more than half of their fluid intake parenterally. Recruitment was conducted prospectively for the 2021–2023 period and retrospectively using database records for the 2018–2021 period. Exclusion criteria were as follows: outborn patients, presence of major congenital malformations, hydrops fetalis inherited disorders of metabolism, suspected pulmonary hypoplasia (based on clinician interpretation of a chest radiograph with small and hypoplastic-appearing lungs), premature rupture of membranes and/or oligohydramnios documented on antenatal ultrasound 3 or more weeks prior to delivery. (Fig. 1).

A local standardised protocol for echocardiographic evaluation of PDA and treatment was used, based on best evidence, and the assessment was usually performed during postnatal day two.^15,16^ The examination was performed by a neonatologist from the functional echocardiography team, who share the same training and follow the same guidelines. The decision to initiate pharmacological treatment was made following a collegial discussion.

Left atrial anterior–posterior dimension and aortic sinus width were measured based on conventional M-mode recordings of the parasternal long- axis, and the LA:AO ratio was calculated. Short axis views revealed the location of the PDA, and the PDA width was measured. Echocardiographic criteria for an hsPDA diagnosis included enlarged left atrial with a LA:AO ratio more than 1.5, unrestrictive pulsatile transductal flow, organ flowmetry compatible with vasoconstriction in the superior mesenteric artery and anterior cerebral artery. The first-line treatment of hsPDA was intravenous Ibuprofen with a three-dose course administered 24 h apart, at a dosage of 10 mg/kg for the first dose and 5 mg/kg for the subsequent doses. Paracetamol was used when there was a relative contraindication to ibuprofen (clinical bleeding, thrombocytopenia < 50,000 platelets/mm mm3, diuresis < 0.6 ml/kg/hour over the last 12 h, uncontrolled sepsis, feed intolerance and in case of NEC suspicion) at a dose of 15 mg/kg intravenously every 6 h for 5–7 days.

A second pharmacological cycle was performed in the occurrence of persistent hsPDA. We administered a second therapeutic cycle of ibuprofen in newborns showing a significant improvement of clinical conditions and/or echographyc findings or indometacin in newborns that showed a slight but not significant improvement of clinical conditions and/or echographyc findings. In case of contraindications a cycle of paracetamol was made.

A third pharmacological cycle was performed still in newborns showing a slight but not significant improvement of clinical conditions and/or echographyc findings, with rescue indometacin or paracetamol based on previously used drugs and the presence of contraindications.

If clinical conditions did not improve or worsened after three cycles of pharmacological treatment, the newborns were referred for surgery in the same hospital or percutaneous closure in an affiliated local hospital.^17,18^

### Data collection

The following data were recorded for each infant: gestational age (GA), birth weight (BW), BW z-score, sex, Apgar score at 5 min, antenatal steroid treatment (complete course), preterm premature rupture of membranes (PROM) > 18 h, diagnosis of clinical chorioamnionitis (defined as maternal fever and one of the following: uterine tenderness, abdominal pain, foul-smelling vaginal discharge, maternal and fetal tachycardia, elevated white blood cell count), and type of delivery.

Additionally, echocardiographic data, pharmacological and nutritional therapies during hospitalization, and complications during hospital stay (respiratory distress syndrome, pneumothorax, pulmonary hemorrhage, necrotizing enterocolitis) were recorded, along with the main postnatal diagnoses included in the secondary outcomes. All collected data were obtained from the clinical records.

### Statistical analysis

All data were collected and stored on a dedicated database. Data are presented as numbers and percentages for categorical variables. Continuous variables are expressed as mean and standard deviation (SD) if they were normally distributed or as median and interquarile range (IQR) for nonnormal data. Comparisons between continuous variables were performed using the *t* test in the case of normal distribution and the Mann-Whitney *U* test in the case of nonnormal distribution. Categorical variables were compared using Fisher exact test or *χ*^2^ with Yates correction. A 2-tailed value of *p* < 0.05 was considered significant. Numbers of pharmacolocital treatments or LA:AO ratio or PDA diametere, non invasive ventilation or length of stay, day of life reaching full enteral feeding, number of days of central line access or weight and head circumference z score at discharge, was analyzed using the Pearson correlation coefficient. Recurrence-free PDA over time was calculated by the Kaplan–Meier method, log-rank test, and Cox regression was used for comparison between groups. The proportional hazards assumption and influential observations for the Cox model were tested.

## Results

### Study population

A total of 82 preterm infants were enrolled: 40 patients in HG and 42 patients in PG. The demographic and clinical characteristics of the participating infants are presented in Tables 1 and 2. The baseline demographics characteristics of the two study groups showed no significant differences (Table 1).

**Table 1:** Baseline demographics of included infants.

Characteristic	Infants, No. (%) (*N* = 82)		*p* value
	Hystorical group (2018-2020) (*n* = 40)	Prospective group (2021-2023) (*n* = 42)	
**Sex**			
Male	14 (35)	19 (45.2)	0.345
Female	26 (65)	23 (54)	
**GA, wk**			
Median (IQR)	26.3 (25–28)	26.0 (25.17–27.3)	0.888
**Twins**	15 (37.5)	18 (42.9)	0.621
**Birth weight**			
Median (IQR), g	732.5 (647.5–953.75)	805 (717.5–1007.5)	0.07
Short for gestational age (<10°pc)	7 (17.5)	4 (9.5)	0.289
**C-section**	9 (22.5)	15 (35.7)	0.189
**Apgar score 1 min**.	5 ± 2	5 ± 2	0.55
**Apgar score 5 min**.	7 ± 1	7 ± 2	0.421
**Antenatal complete steroids courses**	10 (25)	13 (31)	0.55
**PROM**	30 (70)	33 (78.4)	0.702
**Sepsies (n,%)**	26 (65)	25 (58.52)	0.8

*GA* gestational age, *PROM* premature rapture of membrane.

**Table 2:** Comparison of baseline clinical characteristics between historical cohort (2018–2020) and prospective cohort (2021–2023).

	Infants, No. (%) (*N* = 82)		*p* value
	Hystorical group (2018-2020) (*n* = 40)	Prospective group (2021-2023) (*n* = 42)	
**DoL 1° cycle**, mean (SD), d	10.07 ± 8.58	5.14 ± 2.68	**0.001**
**Ibuprophen**	16 (40)	9 (21.43)	0.085
**PDA diameter**, mean (SD), mm	2.32 ± 0.65	2.07 ± 0.70	0.084
**LA: AO**, mean (SD), ratio	1.75 ± 0.36	1.62 ± 0.25	0.091
**SMA absent/retrograde diastolic flow**	18 (45)	11 (26.2)	0.136
**ACA absent/retrograde diastolic flow**	13 (32.5)	2 (4.8)	**0.001**
**Fluid intake 24 h pre PDA treatment,** Mean (SD), ml/kg	125.27 ± 28.33	130.57 ± 47.10	0.563
Intravenous	115.96 ± 32.30	109.71 ± 46.85	0.829
Oral	9.32 ± 22.13	43.08 ± 77.38	0.084
**Fluid resctriction 24 h post treatment** Median (IQR), %	−12 (−5 −20)	0	**<0.01**
**Fluid resctriction 48 h post treatment** Median (IQR), %	−14.3 (−10 −20)	0	**<0.01**
**Fluid intake 24 h post PDA treatment,** Mean (SD), ml/kg	123.66 ± 32.75	148.21 ± 32.82	**0.001**
Intravenous	116.76 ± 35.28	130.26 ± 33.38	0.136
Oral	6.90 ± 22.40	17.94 ± 28.74	0.09
**Diuretics during treatment**	18 (45)	10 (23.8)	**0.015**

*PDA* patent ductus arteriosus, *SMA* superior mesentheric artery, *ACA* anterior cerebral artery, *DoL* day of life, *LA:AO* left atrium to aorta ratio, *SD* standard deviation, *d* days.

Significant differences in Table 2 are reported in bold. No significant differences were observed in echocardiographic parameters at the start of pharmacological therapy, including PDA size, LA:AO ratio, superior mesenteric artery (SMA) flow, and the choice of first-line pharmacological treatment (ibuprofen). (Table 2).

The two groups showed a difference in the timing of treatment initiation (10.07 ± 8.5 days of life in HG and 5.14 ± 2.6 days of life in PG; *p* = 0.001), which was associated with a significant higher number of patients showing impaired organ blood flow in the anterior cerebral artery (ACA), as absent/retrograde diastolic flow at the start of treatment in the HG, treated later respect to the PG, treated earlier, 13 out of 40 (32.5%) *vs* 2 out of 42 (4.8%) respectively, *p* = 0.001 (Table 2).

In infants with hsPDA, fluid intake during the 24 h following the start of treatment was significantly higher in the PG (Table 2), with a greater enteral component, despite treatment starting at an earlier day of life. Regarding fluid intake in the 24 h before treatment initiation (Table 2), this was higher in PG despite being related to fluid intake planned for day 5 of life, compared to HG who received fluid intake planned for day 10 of life, in line with changes in fluid and nutritional management introduced during the period 2021–2023. Moreover the PG was not subjected to fluid restriction after treatment; in contrast, the HG underwent a percentage of fluid restriction after hsPDA treatment, relative to the fluid intake expected for the respective day of life, with a deficit of 12% (IQR 5–20) in the 24 h post-treatment and a deficit of 14.3% (IQR 10–20) in the 48 h post-treatmentas, as shown in Table 2. Similarly, patients in the HG received diuretic therapy more frequently: 18 out of 40 patients (45%) in HG *vs* 10 out of 42 patients (24%) in PG, *p* = 0.01 (Table 2).

### Outcomes

Within the first 2 weeks of life, 8 infants died due to causes unrelated to PDA or its pharmacological treatment for closure and were therefore excluded from the outcome evaluation.

Significant differences in primary and secondary outcomes in Table 3 are reported in bold. We did not observe a favorable effect of fluid restriction on the response to pharmacological treatment for hsPDA closure. This is evidenced by the absence of significant differences in the number of pharmacological cycles required to achieve PDA closure (1.86 ± 0.95 in HG vs. 1.71 ± 1.04 in PG; *p* = 0.73) (Table 3).

**Table 3:** Primary and secondary outcomes.

	Infants, No. (%) (*N* = 74)		*p* value
	Hystorical group (2018–2020) (*n* = 37)	Prospective group (2021–2023) (*n* = 37)	
**Cycles of therapy**	1.86 ± 0.95	1.714 ± 1.042	0.732
**PDA surgical closure**	0	2 (4.8)	0.162
**PTX**	1 (4.76)	7 (17.50)	0.972
**NEC**	7 (17.5)	2 (4.8)	0.075
**IVH 3 prePDA**	7 (17.50)	4 (9.52)	0.327
**IVH 3 postPDA**	11 (27.50)	7 (16.67)	0.278
**PVL**	12 (30)	19 (45.24)	0.10
**ROP**	10 (25)	9 (21.4)	0.702
**BPD**	22 (55)	17 (40.5)	0.244
**Pulmonary hemorrhage**	3 (7.50)	2 (4.76)	0.643
**Mechanical ventilation** mean (SD), d	33 (82.5) 27.57 ± 43.47	34 (81) 15 ± 17.35	0.856 0.1
**Non invasive ventilation** mean (SD), d	37 (92.5) 33.35 ± 30.58	35 (83.3) 41.95 ± 23.84	0.205 0.19
**Inotropes**	17 (42.50)	13 (42.5)	0.35
**Nitric oxide**	11 (27.50)	7 (16.7)	0.284
**Sepsies**	26 (65)	25 (58.52)	0.8
**Days Central Access**, mean (SD), d	61.8 ± 47.8	34.81 ± 27.18	**0.01**
**Day full enteral feeding**, mean (SD), d	61.6 ± 41.96	43.35 ± 26.79	**0.04**
**Head circumference at discharge,** Z score, mean (SD)	−2.4 ± 1.7	−1.9 ± 1.2	0.16
**Weight at discharge** **Z score**, mean (SD)	−2.12 ± 1.29	−1.3 ± 1.2	**0.05**
**Length of stay**, mean (SD), d	108.97 ± 65.4	106.84 ± 52.84	0.87
**Death**	9 (22.5)	4 (10.8)	0.127

*PDA* patent ductus arteriosus, *PTX* pneumothorax, *NEC* necrotizing enterocolitis, *IVH* intraventricular hemorrhage, *PVL* periventricular leukomalacia, *ROP* retinopathy of prematurity, *BPD* Bronchopulmonary Dysplasia, *SD* standard deviation, *d* days.

The two groups did not show differences in the main secondary outcomes (Table 3).

However, the HG showed a higher number of days with a central venous catheter (61.8 ± 47.8 in vs. 34.81 ± 27.18; *p* = 0.01) and a longer time to reach full enteral feeding (61.6 ± 41.96 vs. 43.35 ± 26.79; *p* = 0.04). The mean weight and head circumference, expressed as z-scores, were analyzed at birth and at discharge. The HG had a significantly lower weight z-score at discharge compared to the PG (−2.12 ± 1.29 vs. −1.3 ± 1.2; *p* = 0.05) (Table 3).

**Fig. 2:**
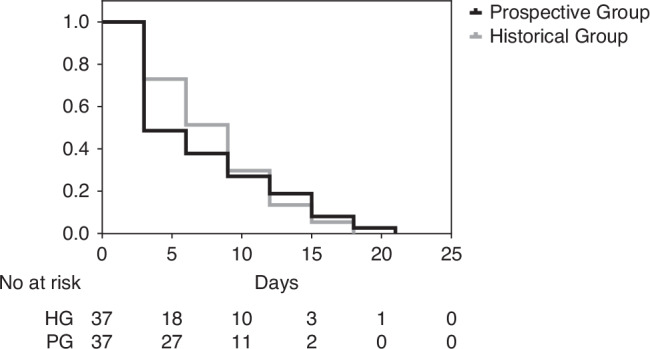
The Kaplan-Meier curve represents the cumulative probability of (PDA) closure over time in preterm with fluid restriction (Historical group—HG) (gray line) and preterm with a fluid intake appropriate to the day of life (Prospective group—PG), (black line). X-axis: Indicates the number of days since the start of treatment until PDA closure. Y-axis: Indicates the cumulative proportion of patients who have not yet achieved PDA closure (survival of the open ductus).

The Kaplan-Meier curve represents the cumulative probability of PDA closure over time in preterm infants with fluid restriction and those without. The two curves show similar trends, suggesting that a fluid restriction had no significant effect on the rate or overall time of PDA closure compared to the group without fluid restriction (Fig. 2).

## Discussion

We assessed the impact of fluid restriction on the efficacy of pharmacological treatment for PDA closure. We compared a PG of patients born in the 2021–2023 period with a HG of patients born in the 2018–2020 period. The HG was subjected to a more restrictive daily fluid intake compared to the study group, with additional fluid restriction during pharmacological treatment for hsPDA. To the best of our knowledge, this is the first study analyzing the impact of fluid restriction during pharmacological treatment for the closure of hsPDA. We demonstrated that fluid restriction does not improve the response to pharmacological treatment for PDA. Indeed, it is associated with an increased number of days with a central venous catheter, a longer time to achieve full enteral feeding, and poorer growth outcomes at discharge, as assessed by weight z-score.

The management of PDA in preterm infants has been a topic of ongoing debate for over 60 years. Some researchers argue that PDA is merely a benign reflection of neonatal immaturity, posing no significant health risks and treating PDA may be unnecessary and potentially harmful. On the other hand, others express concern about the hemodynamic impact of the left-to-right shunt, which increases pulmonary blood flow while reducing systemic perfusion and contributes to serious neonatal complications. Numerous approaches to PDA management have been proposed, ranging from watchful waiting to untargeted prophylactic interventions. However, there is still no clear agreement on which patients should be treated, the optimal timing, or the most effective treatment.

The association between fluid intake volume and the development of symptoms related to PDA in premature infants was first studied in 1977.^6^ Fluid reduction in preterm infants with hsPDA is often considered the first line of treatment. This approach is based on the idea that reducing the shunting in the DA promotes PDA closure by decreasing pulmonary blood flow and mitigating systemic arterial flow reduction, which can lead to organ hypoperfusion and increased risks of IVH, NEC, renal failure, heart failure, and BPD.^19–23^

On the other hand, fluid restriction itself can lead to decreased left ventricular output, reduced systemic blood flow, and an increased risk of dehydration, hypotension, and renal failure.

Among NICUs, there is extreme variability in terms of indications, timing, and modalities of fluid restriction application. This variability, also observed in randomized trials and observational studies, reflects the lack of standardization in this practice and the contrasting evidence regarding its efficacy.

An Italian survey revealed that conservative measures for PDA management are implemented in the majority of NICUs in Italy, with fluid restriction being the preferred approach. In fact, 80% of centers reduce fluid intake in neonates with PDA, though significant variability exists among centers regarding clinical indications and fluid restriction modalities. This variability likely reflects the absence of clear guidelines and recommendations on this topic.^9^ Studies on this subject are limited by the wide variability in the volumes of fluids administered, both in terms of reference standards and the fluid restriction regimens implemented.

A systematic review and meta-analysis of five randomized controlled trials (RCTs) conducted in 2014 concluded that restrictive fluid prescription significantly reduced the risk of PDA and NEC but significantly increased postnatal weight loss.^8^ However, the results of these RCTs were inconsistent, based on relatively short study periods, and largely drawn from studies conducted in the 1980s and 1990s. Caution should therefore be exercised in extrapolating these findings to extremely premature infants, who were underrepresented in these studies.

Similarly, caution should be exercised in interpreting a retrospective study of preterm infants where VLBW neonates had significantly higher percentages of medical unresponsiveness and surgically ligated PDA compared to non-VLBW neonates and the amount of intravenous fluid intake during the first 24 h could independently determine PDA closure.^24^ It is difficult to draw definitive conclusions about the true impact of fluid management on PDA. It is conceivable that physiologically immature infants received increased fluid volumes in response to clinical signs of dehydration, and these infants were already predisposed to worse outcomes due to their immature physiology. Therefore, although excessive fluid intake in the early days cannot be excluded as a potential risk factor for PDA,^7^ increased fluid volume in the first week could simply be a marker of a higher-risk infant.

Numerous studies in recent years have reported the side effects of fluid intake reduction in neonates. De Buyst et al. focused on the hemodynamic impact of fluid intake reduction, identifying in patients subjected to fluid restriction, a reduction in superior vena cava flow, without improvements in PDA closure or respiratory parameters. It has been speculated that a reduction in blood volume could increase systemic vascular resistance, thereby exacerbating the left-to-right shunt through the DA.^25^

Our study demonstrated that patients treated for hsPDA and subjected to fluid restriction did not show a better response to PDA therapy. In addition, infants in the HG had significantly lower discharge weight z-scores, according to the Intergrowth charts. They also required a greater number of central venous catheter days, reflecting the slower advancement of both parenteral and enteral nutrition, and needed more time to reach full enteral feeding compared with infants managed with a higher fluid intake regimen.

Poor postnatal growth in this population may be explained by several factors. Previous studies have reported significantly lower growth in infants with PDA managed with fluid restriction, mainly due to reduced energy and protein intake.^26,27^ Although treated PDA itself is a recognized risk factor for extrauterine growth restriction (EUGR), other determinants—including low gestational age, NEC, and IVH—have also been implicated. Moreover, the duration of parenteral nutrition and the delay in achieving full enteral feeding have been more directly associated with EUGR at NICU discharge, rather than with the mere presence of PDA during hospitalization.^28,29^

In our study, the improvement in achieving full enteral feeding in the second 3-year period, alongside changes in fluid management, was likely supported by the early initiation of minimal enteral feeding (MEF). This was facilitated by the availability of donor human milk for ELBW infants within the first hours of life when maternal milk was not yet available, which enabled the maintenance of a higher proportion of enteral nutrition during PDA treatment, particularly in extremely preterm infants.

A second significant advancement in clinical practice over the past 3 years has been the implementation of bedside functional echocardiography. This allowed for scheduled functional assessments of PDA, enabling treatment before the onset of hemodynamic repercussions. This was demonstrated by a significantly more pathological anterior cerebral artery Doppler flow in the HG.

Fluid management practices for preterm infants with hsPDA differ significantly among neonatal units and are not standardized. Our study does not support the common recommendation of fluid restriction for preterm infants with hsPDA during pharmacological treatment. Fluid therapy should be tailored, also based on daily plasma sodium levels, to meet the infant’s physiological and caloric requirements while ensuring an appropriate balance between systemic and pulmonary blood flow. Furthermore, there is insufficient evidence to indicate that routine use of diuretics promotes ductal closure. Diuretics should only be utilized in cases of evident pulmonary edema or left ventricular dysfunction in neonates with complex hsPDA. The true impact of fluid management on PDA and the physiologic mechanisms that play a role can only be assessed in a randomized control trial to better understand its potential beneficial effects and its potential negative effects such as dehydration, low blood pressure, impaired organ perfusion, and insufficient caloric intake. The present study, however, has certain limitations: notably, its single-center design limited the number of eligible infants, nevertheless, enrolling patients born and discharged from the same center ensures greater uniformity in management; long term neurodevelopmental outcome was not assessed in this study, and future research should address this important aspect. Another limitation is the comparison with a retrospective cohort. However, we are aware that, for ethical reasons, it is currently challenging to compare two groups of patients where one is subjected to fluid management practices that are not aligned with the most up-to-date guidelines.

## Conclusions

The findings from our study demonstrate that fluid restriction does not improve the response to pharmacological treatment for hsPDA. Instead, it is associated with a greater number of days requiring central venous catheters, delayed achievement full enteral feeding, and poorer growth outcomes at discharge. Therefore, our study does not support the standard recommendation of fluid restriction in preterm infants with hsPDA during pharmacological treatment.

## Supplementary information

Supplementary information

## Data Availability

Deidentified data is available from the authors upon request and with appropriate Institutional Review Board approval.
