# Supplementary material for: Standard versus restricted fluid administration in preterm infants undergoing pharmacological treatment for haemodynamically significant patent ductus arteriosus
Source: Pediatr Res. 2025 Oct 27;99(6):2320–6. doi: 10.1038/s41390-025-04497-w (PMC13433318; doi:10.1038/s41390-025-04497-w)
Supplement: Supplementary file 1 [file 41390_2025_4497_MOESM1_ESM.pdf]

Supplemental table S1. ESPGHAN recommendations fluid intake (ml/kg/day) during the first days of life in neonates for weight and day of life:

Postnatal Age	Preterm Neonates >1500 g	Preterm Neonates 1000–1500 g	Preterm Neonates <1000 g
Day 1	60–80 mL/kg/day	70–90 mL/kg/day	80–100 mL/kg/day
Day 2	80–100 mL/kg/day	100–110 mL/kg/day	100–120 mL/kg/day
Day 3	100–120 mL/kg/day	110–130 mL/kg/day	120–140 mL/kg/day
Day 4	120–140 mL/kg/day	130–150 mL/kg/day	140–160 mL/kg/day
Day 5 and onwards	140–160 mL/kg/day	160–180 mL/kg/day	160–180 mL/kg/day

Jochum, F., Moltu, S. J., Senterre, T., et al. ESPGHAN/ESPEN/ESPR guidelines on pediatric parenteral nutrition: Fluid and electrolytes. *Clinical Nutrition, 2018 Dec;37(6 Pt B):2344-2353. doi: 10.1016/j.clnu.2018.06.948. Epub 2018 Jun 18.* https://doi.org/10.1016/j.clnu.2018.06.948

Supplemental table S2. Fondazione Policlinico Universitario A. Gemelli NICU internal protocol (2018 - 2021 years) for fluid management (ml/kg/day) based on gestational age:

Gestational Age	Day 1	Day 2	Day 3	Day 4	Day 5	Day 6	Day 7 and onwards
>26 weeks	60 mL/kg/day	70 mL/kg/day	80 mL/kg/day	100 mL/kg/day	120 mL/kg/day	140 mL/kg/day	150 mL/kg/day
≤26 weeks	70 mL/kg/day	80 mL/kg/day	90 mL/kg/day	110 mL/kg/day	130 mL/kg/day	140 mL/kg/day	150 mL/kg/day
